# Sediment Depth-Dependent Spatial Variations of Bacterial Communities in Mud Deposits of the Eastern China Marginal Seas

**DOI:** 10.3389/fmicb.2018.01128

**Published:** 2018-05-31

**Authors:** Yanlu Qiao, Jiwen Liu, Meixun Zhao, Xiao-Hua Zhang

**Affiliations:** ^1^Laboratory of Marine Microbiology, College of Marine Life Sciences, Ocean University of China, Qingdao, China; ^2^Laboratory for Marine Ecology and Environmental Science, Qingdao National Laboratory for Marine Science and Technology, Qingdao, China; ^3^Key Laboratory of Marine Chemistry Theory and Technology, Ministry of Education, Ocean University of China, Qingdao, China

**Keywords:** bacterial communities, eastern China marginal seas, spatial distribution, diversity, mud deposits

## Abstract

The mud sediments of the eastern China marginal seas (ECMS) are deposited under different hydrodynamic conditions with different organic matter sources. These events have been demonstrated to exert significant influences on microbial communities and biogeochemical processes in surface sediments. However, the extent to which such effects occur in subsurface microbial communities remains unclear. In this study, both horizontal and vertical (five sites, each for eight layers) distributions of bacterial abundance and community composition in mud deposits of the South Yellow Sea (SYS) and East China Sea (ECS) were investigated by quantitative PCR and Illumina sequencing of the 16S rRNA gene. Both bacterial abundance and diversity were higher in the ECS than in the SYS, and tended to be higher in up than in deep layers. *Proteobacteria* (JTB255 marine benthic group), *Acidobacteria* and *Bacteroidetes* were dominant in the upper layers, whereas *Lactococcus*, *Pseudomonas*, and *Dehalococcoidia* were enriched in the deep layers. The bacterial communities in surface and subsurface sediments showed different inter-taxa relationships, indicating contrasting co-occurrence patterns. The bacterial communities in the upper layer samples clustered in accordance with mud zones, whereas those in the deep layer samples of all sites tended to cluster together. TOC δ^13^C and TON δ^15^N significantly affected the bacterial community composition, suggesting that the abundance and composition of organic matter played critical roles in shaping of sedimentary bacterial communities. This study provides novel insights into the distribution of subsurface bacterial communities in mud deposits of the ECMS, and provides clues for understanding the biogeochemical cycles in this area.

## Introduction

Marginal seas are the transitional zones between the coastal and open oceans and occupy about 10% of the global ocean. These shallow, narrow and fast-deposition areas are reported to be major reservoirs of organic carbon burial in the marine system (Hedges and Keil, 1995), and have significant impacts on global biogeochemical cycles and even global climate changes to a considerable degree (Chen C.T.A. et al., 2004; Coelho et al., 2013). Correspondingly, sediments in these areas harbor a higher abundance of microbes than those in open oceans (Kallmeyer et al., 2012). These abundant microorganisms contribute significantly to the cycling of marine biogenic elements (Azam and Malfatti, 2007; Falkowski et al., 2008), especially carbon (Dyksma et al., 2016).

Bacterial communities were found to vary in different marginal sea sediments (Bowman and McCuaig, 2003; Bertics and Ziebis, 2009; Harrison et al., 2009; Zinger et al., 2011; Wang et al., 2012; Learman et al., 2016; Probandt et al., 2017). These can be explained by environmental heterogeneity (including sediment sources and hydrodynamic conditions) that can significantly influence the distribution of microbial communities and related biogeochemical processes. For example, organic matter has been demonstrated to be a driver of benthic microbial community structure across the Antarctic surface sediment (Learman et al., 2016). Meanwhile, shifts in bacterial community were observed in oil-contaminated and nitrogen-polluted sediments in the Antarctic near shore, Mediterranean Sea and East China Sea (ECS) (Powell et al., 2003; Polymenakou et al., 2006; Xiong et al., 2014), suggesting composition of organics and nutrients to be important factors as well. In addition, dissolved oxygen (DO) of the bottom water, temperature and sediment median grain size, have been detected to be vital factors shaping benthic bacterial communities in the Chinese marginal sea (Bohai Sea and Pearl Estuary), Arctic and North Sea (Wang et al., 2013; Liu et al., 2014; Zheng et al., 2014; Nguyen and Landfald, 2015; Probandt et al., 2017). Most studies mentioned above were based on surface sediments, but relatively few have focused on the vertical profile of bacterial communities in typical marginal sediments (Franco et al., 2007; Böer et al., 2009; Harrison et al., 2009; Liu et al., 2014). Depth related shifts in bacterial community in marginal sediments have been reported and were attributed to different factors such as contents of organic carbon, chlorophyll *a* and inorganic nutrients (Bowman and McCuaig, 2003; Böer et al., 2009; Harrison et al., 2009). None of these studies included a highly resolved vertical profile of sedimentary bacterial communities. Thus, the extent to which the benthic surface environmental heterogeneity affect subsurface microbial communities needs further investigation.

The eastern China marginal seas (ECMS) are typical eutrophic seas with different mud areas formed by sediments derived mainly from the Yellow River and Yangtze River. These mud areas are characterized by different sediment sources and hydrodynamic conditions resulted from complex water masses and ocean currents; therefore, they provide different environmental niches for microorganisms to survive. Accordingly, previous studies have shown distinct distribution patterns of functional microorganisms in different ECMS mud sediments (Yu et al., 2016; Gao et al., 2017). However, compositional distributions of total bacterial community in different mud sediments are currently unknown. We hypothesized that the total bacterial communities varied in surface sediments but converged in subsurface sediments in different mud areas of the ECMS. In this study, a high resolution vertical profile of bacterial abundance and community composition from five sites, each for eight layers, of the ECMS was provided. In addition, the bacterial co-occurrence patterns, which can help uncover potential inter-taxa relationships, in both surface and subsurface sediments were explored by using correlation based network analysis.

## Materials and Methods

### Study Site and Sampling

To compare sedimentary bacterial communities in different mud zones of the ECMS, five sites (SYS01, SYS02, ECS01, ECS02, and ECS03) distributed in four typical mud zones of the South Yellow Sea (SYS) and ECS were chosen. Locations of these samples have been reported by Yu et al. (2016). SYS01 and SYS02 are located in the SYS mud zone, where the deposits are mainly from sinking of the modern and old Yellow River-derived sedimentary organic matter (Hu et al., 2013). In addition, mud deposits in this area are considered as a result of the presence of cold water mass in summer, accompanied by seasonal weaken of the Yellow Sea Warm Current (Hu, 1984). Situated in the Yangtze River Estuary mud zone, ECS01 is mainly influenced by freshwater flowed out of the Yangtze River, which makes Yangtze River to be the dominant sediment source of ECS01 (Liu et al., 2007). ECS02 is located in the Zhe-Min mud zone. This area is influenced by a couple of alternatively predominant reversed currents (the Zhe-Min Coastal Current and Taiwan Warm Current); its sediments are mainly transported from the Yangtze River and the estuary mud zone along the Zhe-Min coast (Liu et al., 2007). ECS03 belongs to the distal Cheju Island mud zone, and its sediments are derived from both the Yangtze River and the old Yellow River, transported by the Yellow Sea Warm Current and river runoff from the Yangtze River (Liu et al., 2003).

The sediment samples were collected by a box corer during a cruise of R/V *Dong Fang Hong 2* from 12 July to 2 August, 2013. Two PVC tubes were used to subsample the collected sediments at each site. One PVC core was immediately sliced at a 1-cm interval with a stainless-steel cutter and the sliced sediments were stored at -20°C (onboard) or -80°C (in laboratory) before organic matter measurement and DNA extraction. An aliquot of sediments at depths of 0–1, 12–13, and 32–33 cm from sites SYS01, SYS02, ECS02, and ECS03 was fixed with paraformaldehyde (2% final) in sterile plastic vessels and conserved in 1:1 PBS-ethanol at -20°C for 4′, 6-diamidino-2-phenylindole (DAPI) counting. The parallel core was prepared for pore water extraction. Pore water samples were collected by the Rhizon samplers at the cm-scale, poisoned by HgCl_2_ and stored at 4°C before dissolved inorganic nutrient measurement. For each core, eight sediment layers that were 0–1, 1–2, 2–3, 3–5, 7–8, 12–13, 22–23, and 32–33 cm (written as -0, -1, -2, -3, -5, -10, -20, and -30 cm, respectively) were chosen for microbiological analyses. Total organic carbon (TOC), total nitrogen (TN), stable carbon (TOC δ^13^C) and nitrogen isotopes (TON δ^15^N) in sediments, dissolved inorganic nutrients (NO_3_^-^, NO_2_^-^, NH_4_^+^, PO_4_^3-^, SiO_3_^2-^ and SO_4_^2-^) in pore water, salinity, DO and Chl *a* in bottom water were determined as previously described (Yu et al., 2016).

### DNA Extraction

Genomic DNA was extracted from 0.25 g of sediment (wet weight) using the Power Soil DNA Isolation Kit (Mo Bio Laboratories, Inc., Carlsbad, CA, United States) and a FastPrep-24 cell disrupter (MP Biomedicals, Irvine, CA, United States) according to the manufacturer’s instructions. Quality and quantity of the extracted DNA were measured by a Nanodrop spectrophotometer ND-2000 (Thermo Fisher Scientific, United States). DNA was then subpackaged and stored at -80°C.

### Quantification Analysis

Paraformaldehyde fixed sediment samples were diluted and homogenized with low-power ultrasonic wave at 20 W for 30 s. A volume of 50 μl sonicated sample was mixed with 10 mL PBS, collected on the 0.2-μm pore size filter (Isopore GTTP, Millipore), and stained with DAPI. For each sample, cell numbers were counted in ten random views under the fluorescence microscope. To exclude eukaryotic cells, only cells that are 0.5–5 μm in size were counted.

Quantitative PCR was performed to quantify the abundance of total bacteria and sulfate-reducing bacteria (SRB) in the samples using primers of the 16S rRNA gene and dissimilatory sulfite reductase β-subunit (*dsrB*) gene, respectively (Varon-Lopez et al., 2013; Yin et al., 2013). A 20 μl mixture contained 10 μl of SYBR Premix ExTaq II (2×), 0.4 μl of ROX Reference Dye II (50×) (TaKaRa, Tokyo, Japan), 0.2 μl of primers for each gene (10 μM), and 2 μl of template. Primers and thermal cycling steps are shown in **Table 1**. All assays were conducted in triplicate with negative controls using an ABI 7500 Real-Time PCR System (Applied Biosystems, Foster City, CA, United States).

**Table 1:** Primers and PCR conditions used for the PCR amplification.

Target gene	Primer name and sequence (5′–3′)		Thermal profile	Reference
Bacterial 16S rRNA	Eub338F	ACTCCTACG GGAGGCAGCAG	30 s at 95°C, followed by 40 cycles of 30 s at 94°C, 30 s at 53°C, and 30 s at 72°C (for q-PCR)	Yin et al., 2013
	Eub518R	ATTACCGCGGCTGCTGG		
	Eub515F	GTGCCAGCMGCCGCGG	2 min at 95°C, followed by 25 cycles of 30 s at 94°C, 30 s at 55°C, and 30 s at 72°C, then, 5 min at 72°C (for sequencing)	Xiong et al., 2012
	Eub907R	CCGTCAATTCMTTTRAGTTT		
SRB *dsrB*	DSRp2060F	CAACATCGTYCAYACCCAGGG	30 s at 95°C, followed by 40 cycles of 30 s at 94°C, 30 s at 55°C, and 30 s at 72°C	Varon-Lopez et al., 2013
	DSR4R	GTGTAGCAGTTACCGCA		


Standard curves were constructed by PCR amplifying a 10-fold serial dilution of plasmids containing target gene fragments. The amplification curves showed well linear relationships (*R*^2^> 0.999) and the amplification efficiencies were 91.65 and 95.28% for the bacterial 16S rRNA gene and *dsrB* gene, respectively. The single-peak melting curves and the only bond in gel electrophoresis guaranteed specificity of the qPCR analysis.

### High Throughput Sequencing and Reads Processing

The PCR and sequencing were performed as previously described (Liang et al., 2015) with minor modifications. Primers Eub515F/Eub907R (**Table 1**) were used for bacterial 16S rRNA gene amplification. The forward and reverse primers were tagged with adapter, pad and linker sequences, and the reverse primer was linked with barcode sequences for pooling of multiple samples in one run of MiSeq sequencing. PCR was run on an ABI GeneAmp^®^ 9700 cycler and thermal cycling steps are shown in **Table 1**. The PCR products of each sample were pooled, purified by an AxyPrep^TM^ DNA Gel Extraction Kit (Axygen, Hangzhou, China) and quantified using a QuantiFluor^TM^-ST Solid Standard (Promega, Madison, WI, United States). Sequencing was conducted on a MiSeq Desktop Sequencer at Majorbio Bio-Pharm Technology Co., Ltd., Shanghai, China.

The raw data were filtered according to the pipeline of Quantitative Insights into Microbial Ecology (QIIME^1^, Caporaso et al., 2010). Reads were assigned to samples according to their barcodes with no mismatch. The raw reads that had a quality score higher than 20 over a 5 bp window size and a minimum length of 100 bp (Kong, 2011) were retained. The pair-end reads were joined with at least a 50 bp overlap and less than 5% mismatches using FLASH (Magoc and Salzberg, 2011). A perl script daisychopper.pl (Gilbert et al., 2009) was used to randomly subsample sequences from each sample according to the least read numbers for equalizing sampling efforts. Operational taxonomic units (OTUs) clustering and taxonomy assignment were also performed in QIIME. Specifically, OTUs were defined at a 97% sequence similarity level, and then chimera sequences were detected and removed with UCHIME (Edgar et al., 2011) as recommended by QIIME tutorials. Taxonomy was assigned using the RDP Classifier v2.2 (Wang et al., 2007) against the SILVA v115 16S rRNA gene reference database^2^ with a minimum support threshold of 70%.

The Illumina sequences were deposited in the National Center for Biotechnology Information Short Read Archive database under SRP076973.

### Statistical Analysis

The diversity indices, including Good’s coverage, Chao1 and Shannon index, were calculated for alpha diversity analysis. Molecular ecological network analyses were conducted by the package ‘Hmisc,’ ‘igraph,’ and ‘qvalue’ in R software (RDC TEAM, 2008) using bacterial groups at the family level with read numbers > 50 across all samples to simplify the network. Co-occurrence pairs with a Spearman’s correlation coefficient > 0.7 or < -0.7 and a *P*-value < 0.01 (Benjamini and Hochberg adjusted) were considered as a valid co-occurrence event. The R script was provided in Supplementary Material. Gephi (version 0.8.2 beta, Bastian et al., 2009) was used for network visualization. Linear discriminate analysis (LDA) effect size (LEfSe) (Segata et al., 2011) was used to identify taxa with significant differences between mud zones and depths at various taxonomic levels. For beta diversity, classification of bacterial communities was performed by principal coordinate analysis (PCoA) using Fast UniFrac (Lozupone et al., 2011). Pairwise analyses of similarities (ANOSIM) was performed in PRIMER 5 (Plymouth Marine Laboratory, West Hoe, Plymouth, United Kingdom). The relationships between phylotypes and environmental factors were evaluated by redundancy analysis (RDA) in CANOCO (Version 5.0, Microcomputer Power) with 9999 Monte Carlo permutation tests using square root-transformed data. Pearson correlation test was also used to evaluate correlations between percentage composition of taxa and environmental factors. In order to accurately estimate the correlations, only the top 20 phyla, top 30 classes, top 50 orders, top 50 families, top 50 genera were tested.

## Results

### Environmental Characterization

Detailed sediment and pore water environmental parameters have been described by Yu et al. (2016). In brief, at all sites, the NO_3_^-^ concentration in pore water, TOC and TN content in sediment had a tendency to decrease with depth. Higher sedimentary *C*/*N* ratio and NO_3_^-^ concentration were detected in SYS than in ECS samples. The pore water concentration of NH_4_^+^ at ECS01 was higher than that at other sites. Much depleted values of TON δ^15^N and TOC δ^13^C were observed in sediment samples of ECS01.

### Direct Cell Counting and Quantitative PCR

**FIGURE 1:**
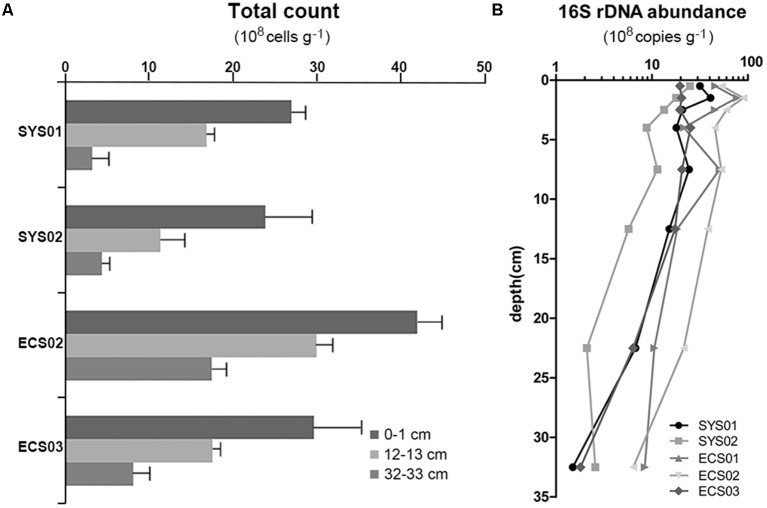
Total cell counts **(A)** and 16S rRNA gene abundance **(B)** of sediment cores from the Eastern China Marginal Seas.

The result of direct cell counting showed that microbial cell numbers in the ECMS varied from 3.17 × 10^8^ to 4.19 × 10^9^ cells g^-1^ (**Figure 1A**). The microbial cell numbers decreased with depth, and significant difference was observed between 0–1 cm samples and 32–33 cm samples (*P* < 0.05). Meanwhile, sediments from the ECS contained more microbial cells than those from the SYS (*P* < 0.01).

Quantitative PCR was also used to detect the abundance of the bacterial 16S rRNA gene, which showed a range of abundance between 1.5 × 10^8^ (SYS01-30) to 9.0 × 10^9^ copies g^-1^ (ECS02-1) (**Figure 1B**). Similar to the result of direct cell counting, samples from sites located on the south tended to have higher copy numbers of the bacterial 16S rRNA gene. Vertically, the abundance fluctuated at a high level within the top 5 cm, and then declined gradually with depth. The 16S rRNA gene abundance was significantly correlated with PO_4_^3-^ (*r* = -0.466, *P* = 0.007), TN (*r* = 0.463, *P* = 0.003), TON δ^15^N (*r* = -0.439, *P* = 0.005), and *C*/*N* (*r* = -0.321, *P* = 0.043). Copy number of *dsrB* gene varied in the range of 2.7 × 10^6^ to 9.6 × 10^7^ copies g^-1^, and no significant difference was observed among studied sites or depths.

### Bacterial Diversity and Richness

A total of 1,472,593 overlapped reads across the 40 samples were generated through Illumina sequencing, and 1,190,856 reads were left after quality control (Supplementary Table S1). Read numbers in each sample were limited to 24,048 after rarefaction for further analyses. All sequences yielded 10,746 OTUs at a 97% sequence similarity level (Supplementary Data Sheet S2). The Good’s coverage values ranged from 91.99 to 98.17% across samples, indicating that sequences generated from these samples could represent most of the bacterial community in the studied sites. Sites located in the ECS had higher Chao1 and Shannon indices than those in the SYS (*P* < 0.05). Site ECS03 located in the Distal Cheju Island mud area had the highest bacterial diversity in terms of Shannon index (*P* < 0.05). As for depth, Shannon diversity of the surface samples (0–2 cm) was higher than that of the 30 cm samples (*P* < 0.05). A negative correlation was found between Shannon diversity and *C*/*N* (*r* = -0.544, *P* = 0.0003).

### Taxonomic Description

In total, 47 phyla were observed in the 40 ECMS sediment samples. The most dominant phylum was *Proteobacteria* occupying 45.62% of all sequences. This was followed by *Planctomycetes*, *Chloroflexi*, *Acidobacteria*, *Bacteroidetes*, *Firmicutes*, *Nitrospirae*, candidate division WS3, *Gemmatimonadetes* and *Actinobacteria*, which jointly accounted for 44.43% of all sequences. Within *Proteobacteria*, *Deltaproteobacteria* (21.93%) and *Gammaproteobacteria* (18.79%) were the most abundant classes. In addition, eight minor phyla (SM2F11, WCHB1-60, OC31, CKC4, candidate division KB1, GOUTA4, *Thermotogae*, and GAL08) were represented each by less than 50 sequences.

**FIGURE 2:**
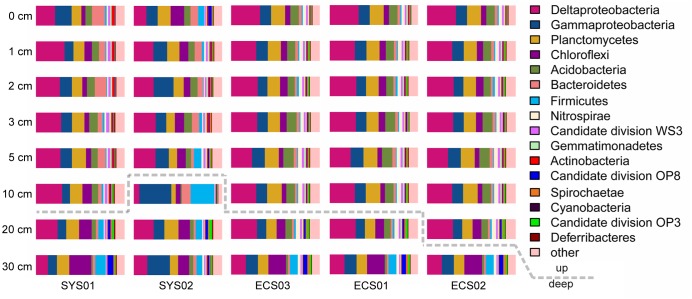
Relative abundance of dominant phyla (*Deltaproteobacteria* and *Gammaproteobacteria*) in different sediment samples. Dotted line divided the two groups defined by the Bray–Curtis similarity analysis at the genus level shown in Supplementary Figure S1.

**FIGURE 3:**
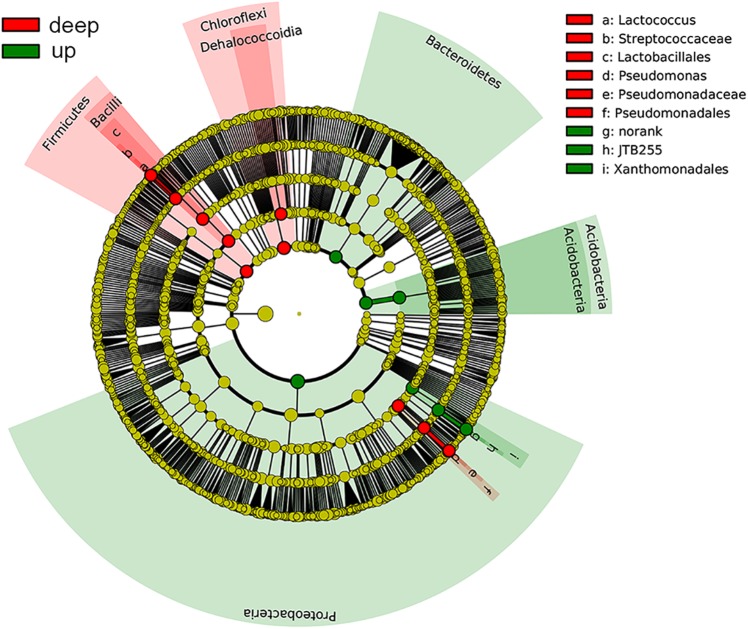
Cladograms showing the differences in relative abundance of bacterial groups between up and deep layers according to the LEfSe analysis with a LDA threshold of 3.5. Taxa with significant differences were highlighted by colored circles and shadings.

The bacterial community compositions varied among depths and sites (**Figure 2**). The proportions of *Proteobacteria*, *Acidobacteria*, and *Bacteroidetes* were high in surface sediments, whereas *Chloroflexi* and *Firmicutes* tended to be enriched in deep layers. The sample similarity analysis based on the Bray–Curtis dissimilarity at the genus level showed that samples were clustered into two groups (Supplementary Figure S1). The boundary between these two groups was plotted as a dotted line in **Figure 2**, illustrating the separation of surface and deep bacterial communities. To discover bacterial groups with significant differences between these two sediment types, LEfSe was conducted from the phylum to genus levels with a LDA threshold of 3.5. The results of LEfSe confirmed the tendency shown at the phylum and genus levels and revealed that *Lactococcus* (genus of *Firmicutes*, dominated by *Lactococcus piscium*), *Pseudomonas* (genus of *Pseudomonadales*, dominated by *Pseudomonas azotoformans* and *P. fragi*) and *Dehalococcoidia* (class of *Chloroflexi*) were significantly abundant in deep layers, whereas *Acidobacteria* (class) and JTB255 marine benthic group (JTB255-MBG, a family of *Xanthomonadales*) preferred surface layers (**Figure 3**). In addition, LEfSe with a LDA value of 3.5 was also used to predict effect differences in bacterial groups among different sites (Supplementary Figure S2). A total of 28 bacterial groups, including five phyla, five classes, six orders, seven families, and five genera, were highlighted to be the specialized taxa for each site. *Syntrophobacterales* from the family to genus levels and *Desulfobacteraceae* at the family level were enriched at SYS01. The abundance of *Firmicutes*, *Chloroflexi*, and *Pseudomonadales* were significantly higher at SYS02. JTB255-MBG was enriched at ECS01, making the proportion of *Proteobacteria* reached to its top at this site. *Bacteroidetes* (from phylum to class levels) was enriched at ECS02. ECS03 had a higher abundance of *Planctomycetes* and *Deltaproteobacteria* than other sites.

**FIGURE 4:**
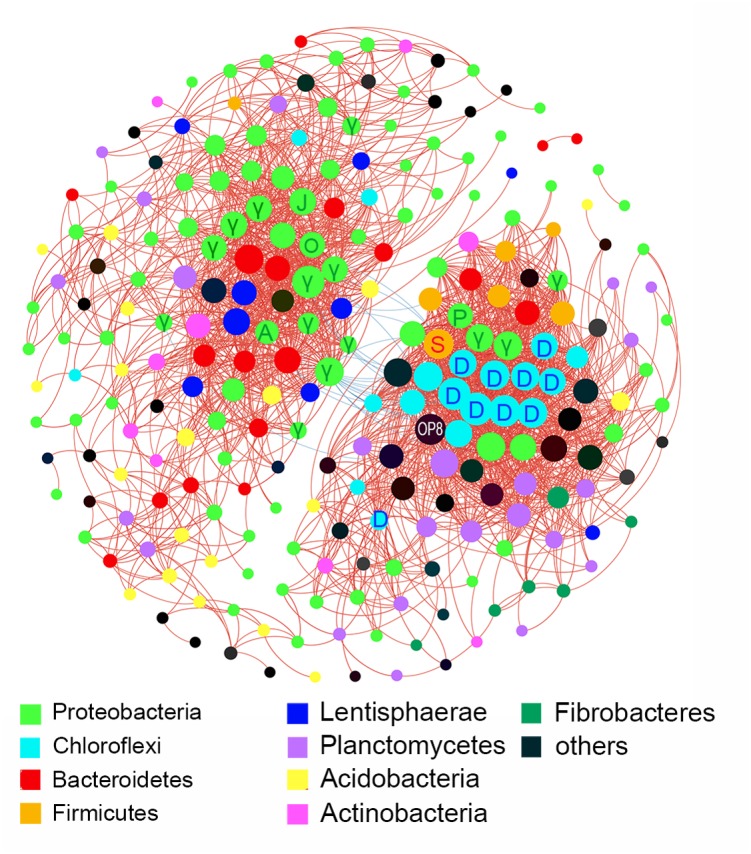
Inter-taxa relation network with thresholds of ±0.7 for Spearman’s coefficient and 0.01 for *P*-value. Correlations between families were represented by colored lines between nodes (red for positive, blue for negative). Size of node depended on the number of connections. A, *Alteromonadaceae*; D, *Dehalococcoidia*; γ, *Gammaproteobacteria*; J, JTB255; O, *Oceanospirillaceae*; P, *Pseudomonadaceae;* S, *Streptococcaceae.*

Inter-taxa relationship network was structured based on bacterial families whose read numbers were more than 50 across all samples. A total of 242 nodes and 2289 edges were presented in the network with a threshold of ±0.7 for Spearman’s coefficient and 0.01 for *P*-value (**Figure 4**). The bacterial families from *Proteobacteria*, *Acidobacteria*, and *Planctomycetes* displayed wide correlations with others. *Proteobacteria* made up more than 1/3 of the nodes in the network. The families tended to networking into two modules, and hubs of the two modules belonged to *Proteobacteria*, *Bacteroidetes*, *Lentisphaerae*, and *Chloroflexi*, and *Proteobacteria*, *Firmicutes*, and *Planctomycetes*, respectively. Twenty-six negative correlations were observed and distributed mainly between *Proteobacteria* and *Chloroflexi*.

A total of 36 pairs of bacterial groups and environmental factors were observed to have significant correlations (*P* < 0.01 and |*r*| > 0.6) (Supplementary Table S2). Sixteen taxa belonging to *Chloroflexi, Planctomycetes, Spirochaetae*, candidate division OP8 and *Deltaproteobacteria* were significantly correlated with sediment depth. Nine taxa belonging to *Deltaproteobacteria*, *Gammaproteobacteria* and BD2-11 terrestrial group (order of *Gemmatimonadetes*) and seven taxa belonging to *Bacteroidetes, Nitrospirae*, and *Planctomyces* showed significant correlations with TON δ^15^N and TOC, respectively.

### Community Comparison at the OTU Level and Environmental Factors Explaining Community Variations

**FIGURE 5:**
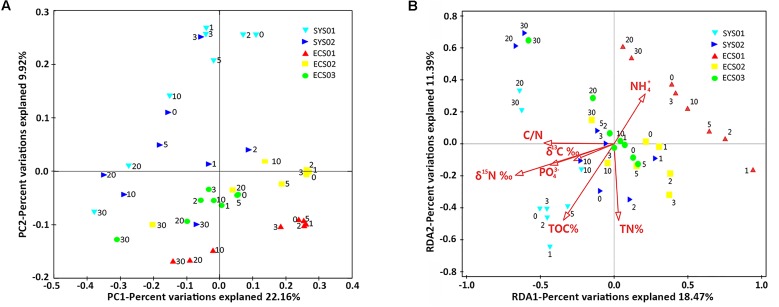
Community analysis at the OTU level. **(A)** Unweighted PCoA plot with PC1 and PC2. **(B)** RDA analysis illustrating the relationship between bacterial community at the OTU level and top environmental variables.

The samples clustered basically according to different mud zones as shown in the PCoA (**Figure 5A**), which considered both the topology of evolutionary trees and abundance of OTUs. The upper layer samples clustered in accordance with mud zones, whereas the deep layer samples of all sites tended to cluster together. To be specific, the upper layer samples (0, 1, 2, 3, and 5 cm) of each site clustered tightly with the exception that those at SYS02 were slightly scattered. Contrastingly, the deep layer samples (20 and 30 cm) of each site were more similar with each other. Interestingly, sediments at the 10-cm layer displayed different clustering relationships in different sites. At ECS02 and ECS03, the 10-cm layer resembled more closely the upper layer samples of the same site, whereas at SYS01, SYS02, and ECS01, they tended to group with the deep layer samples. The two-way ANOSIM revealed that sediment depth (global *R* = 0.835, *p* < 0.001) could explain more variances than mud zones (global *R* = 0.690, *p* < 0.001). RDA analysis was performed and uncovered that seven environmental factors had significantly influences, which jointly accounted for 70.2% of the total variation. TOC δ^13^C (*F* = 5.4, *P* = 0.001) contributed the most with 29.0%, followed by TON δ^15^N (*F* = 5.2, *P* = 0.001), TOC (*F* = 4.3, *P* = 0.001), PO_4_^3-^ (*F* = 3.1, *P* = 0.002), NH_4_^+^ (*F* = 2.3, *P* = 0.01), TN (*F* = 2.3, *P* = 0.017), and *C*/*N* (*F* = 2.3, *P* = 0.023). No significant correlation was observed between NO_3_^-^, NO_2_^-^, or SiO_3_^2-^ and the communities. Influences of the top seven environmental factors on bacterial communities were shown in **Figure 5B**. NH_4_^+^ seemed to exert significant impacts on structuring bacterial communities of ECS01. The up sediments of SYS01 appeared to be separated from other samples by TOC.

## Discussion

### Bacterial Abundance in Sediments of the ECMS and Potential Environmental Drivers

In this study, direct cell counting was implemented to evaluate the microbial abundance in sediments of the ECMS. According to Liu et al. (2015), the bacterial abundance was two to three orders of magnitude higher than the archaeal abundance in sediment of the ECMS. Thus, the counted cell numbers are approximately equal to the bacterial cell numbers. The cell counts were consistent with the result of 16S rRNA gene quantification. Sedimentary bacterial abundance in the ECMS was in the same range with that reported in the SYS (Liu et al., 2015), and was slightly lower than that in bioturbated coastal sediments from the Catalina Island (Bertics and Ziebis, 2009; Plotieau et al., 2013). By contrast, this abundance was higher than that in the Eastern Mediterranean Sea (Polymenakou et al., 2006) and in ODP sites from the Okinawa Trough and Peru margin (Mauclaire et al., 2004; Jean et al., 2005). Different environmental features may explain some of this variance in bacterial abundance across different areas. The bacterial abundance in sediments from the southern mud areas especially at site ECS01 and ECS02 was significantly higher (**Figure 1B**). These two sites are located just outside of the Yangtze River Estuary and Zhe-Min coasts and are readily influenced by terrigenous nutrient input from land, which could lead to the observed high bacterial abundance.

The abundance of bacteria steadily decreased with sediment depth, in well agreement with the global distribution pattern of benthic microbial abundance (Kallmeyer et al., 2012). Generally, aerobic bacterial respiration consumed DO rapidly in upper layers of the eutrophic sediment, which would result in reduced rate of microbial carbon oxidation in deep sediments and subsequent decrease in bacterial abundance (Røy et al., 2012). Availability of organic matter, as the main electron donor in marine sediments, may also affect bacterial abundance. In this study, the 16S rRNA gene abundance was positively correlated with TN (*P* < 0.01), negatively correlated with *C*/*N* (*P* < 0.01) and TON δ^15^N (*P* < 0.05). These results could partly contribute to the negative correlation between Shannon diversity and *C*/*N*, and revealed that the content, source and composition of organic matter are important in determining the abundance of benthic bacteria in the ECMS, with fresh and marine organic matter (higher TN and lower *C*/*N* ratio) supporting higher bacterial abundance.

The abundance of *dsrB* examined in this study was similar to that in the Pearl River estuary (Jiang et al., 2009), Bohai Sea and Yellow Sea (Liu et al., 2015), but lower than that in the Baltic Sea (Leloup et al., 2009) and Blake Sea (Leloup et al., 2007). As sulfate-reducing prokaryotes are anaerobic, the high level of DO in overlaying water of the ECMS sediments, compared with that in the Baltic Sea and Black Sea, may contribute to these variations. No significant differences in copy numbers of *dsrB* gene were observed among studied sites or depths. This was consistent with the invariable SO_4_^2-^ contents observed in the pore waters (Yu et al., 2016).

### Distribution Patterns of Bacterial Community in Sediments of the ECMS and Potential Environmental Drivers

Limited studies of benthic microbial community in the ECMS were focused only on surface sediments. They showed that sediment sources, hydrodynamic conditions and concentration of nutrients might be the crucial factors in shaping bacterial communities (Liu et al., 2014; Xiong et al., 2014). To uncover the extent of influence of such effects on subsurface bacterial communities, the vertical profile of bacterial communities in mud sediments of the ECMS was sampled in this study. We found that bacterial communities of the up and deep sediment layers in the ECMS exhibited contrasting distribution patterns across sites.

Bacterial communities of the upper layers in each site were clearly separated (**Figure 5A**) and were found to be influenced by different environmental factors, such as TOC, NH_4_^+^ and PO_4_^3-^ (**Figure 5B**). TOC separated the upper layer sediment of SYS01 from others, confirming the role of TOC as an important factor in shaping relative abundance of benthic bacterial groups (Jorgensen et al., 2012; Liu et al., 2014). NH_4_^+^ distinguished the bacterial communities in ECS01 sediments especially the upper layer samples from others, confirming that sedimentary bacterial community structures can be effected by nitrogen pollution (Xiong et al., 2014).

These distribution patterns are reflected by differential correlations between taxonomic groups and environment factors, in particular organic matter and nutrients (Supplementary Table S2). For example, *Flavobacteriaceae*, a major group of *Bacteroidetes*, showed a significantly positive relationship with TOC (*r* > 0.6 and *P* < 0.01), and this agreed with their chemoorganotrophic lifestyle functioning especially in degrading high molecular weight dissolved organic matter, such as polysaccharides (Bennke et al., 2016; Teeling et al., 2016). *Planctomyces* also preferred high concentration of nitrogen and organic carbon substrates. However, *Nitrospirae* preferred relatively oligotrophic environments as evidenced by the significantly negative relationship with TOC. *Nitrospirae* are nitrite oxidizing bacteria functioning in aerobic nitrite oxidization. However, they have been found to widely distribute in anaerobic marine sediments (Liu et al., 2014; Nunoura et al., 2016; Chen et al., 2017). Whether sedimentary *Nitrospirae* are inactive or have other uncharacterized physiologies needs further investigation. JTB255-MBG, *Acidiferrobacter* and BD2-11 terrestrial group (belonging to *Gemmatimonadetes*) preferred substrates with a low content of TON δ^15^N, while *Syntrophobacterales* was opposite. These results confirmed that varied sediment sources could provide different environmental niches for the growth of different bacterial communities.

Different from the scattered distribution of upper layer samples, the deep layer (20 and 30-cm layer) samples showed a closer clustering relationship regardless of studied sites (**Figure 5A**). TN and TOC seemed to play roles in converging the deep layer samples of different mud zones (**Figure 5B**). However, it was noteworthy that these environmental factors fluctuated more widely among sites than among depths of one site. Thus, they might be not the direct driving force that clustered the deep layer samples of studied sites. As mentioned above, these factors mainly influenced the relative abundance of up sediment-dominant *Flavobacteriaceae*, JTB255-MBG and *Acidiferrobacter*. These impacts would disappear in deep sediment layers with a lower abundance of up sediment-dominant taxa. Subsequently, the deep layer samples clustered closely. Indeed, DO under the top 1 μm sediments decreased sharply from ∼120–250 μM to an undetectable level in the study area (Yu et al., 2016). Thus, it might be inferred that DO, or redox state, was the crucial factor contributing to the differences between up and deep layer communities.

It was interesting to note that the 10 and 20-cm layer samples displayed different clustering patterns in each site. We speculated that this discrepancy might be attributed to site-specific hydrodynamic conditions, although no environment factor detected here could explain this difference. For example, ECS02 was influenced by the Taiwan Warm Current, resulting in a higher summer flow velocity in this site (Lim et al., 2007; Liu et al., 2007), while SYS02 was located in the bottom of the Yellow Sea Trough covered by the Yellow Sea Cold Water Mass, causing a lower flow velocity and deposition rate than other sites detected (Huh and Su, 1999; Yang et al., 2003; Chen Z. et al., 2004). These differences in fluid dynamics lead to varied particle size and redox profiles across different sites, thus influencing the cluster of 10 and 20-cm layer samples.

### Bacterial Community Compositions in Sediments of the ECMS

The sedimentary bacterial community composition in the ECMS was in high accordance with previous studies of the same area (Xiong et al., 2014; Liu et al., 2015) and other marginal seas (Zinger et al., 2011; Wang et al., 2012; Sun et al., 2013). In comparison with those from the deep sea or coastal areas adjacent to open oceans (Schauer et al., 2010; Dyksma et al., 2016; Walsh et al., 2016), a higher ratio of *Deltaproteobacteria* to *Gammaproteobacteria* was observed in all sediment depths of this study. *Deltaproteobacteria* and *Gammaproteobacteria* (average 48.1 and 41.2%, respectively) were the major predominant classes of *Proteobacteria*, and they have been demonstrated to play important roles in organic matter mineralization and dark carbon fixation, respectively, in coastal sediments (Thamdrup and Canfield, 1996; Dyksma et al., 2016). The high abundance of *Deltaproteobacteria* relative to *Gammaproteobacteria* may reflect specific response of marginal bacterial communities to terrigenous organic inputs, and indicate a higher potential of organic matter mineralization than carbon fixation in the ECMS sediments.

There were significant differences in diversity and composition of bacterial communities between up and deep sediment samples (Supplementary Figure S1 and **Figure 5A**). The upper layers owned a more diverse community than the deep layers. Species capable of thriving under aerobic and anaerobic environments can coexist at the shallow sediment, thus resulting in the high diversity. LEfSe analysis showed that *Lactococcus* and *Pseudomonas* of *Gammaproteobacteria*, and members of *Dehalococcoidia* (class of *Chloroflexi*) were enriched in the deep sediment samples (**Figure 3**). The former two genera were usually found in non-marine environments and played significant roles in spoilage of meat, dairy and fish (Williams et al., 1990; Champagne et al., 1994; Sakala et al., 2002). The proportions of these two genera were higher in several deep layer samples, which might be related to the general decrease in bacterial abundance with depth combined with their presence as contaminants in the extraction kit (Salter et al., 2014). However, sampling and DNA extraction methods in the present study were in accordance with methods used in previous studies on different samples (Nguyen and Landfald, 2015; Chen et al., 2017), in which case these potential contaminant genera were not detected. Thus, it is also possible that they might exist in deep layer sediments and feed on organics, such as remnants of marine animals. *Dehalococcoidia* were widely distributed in deep marine sediments (Durbin and Teske, 2011; Jorgensen et al., 2012). They exhibited significant correlations among each other and formed the deep-abundant module in the network analysis illustrating inter-taxa relationships (**Figure 4**). Different subgroups of *Dehalococcoidia* can inhabit different ecological niches (Bowman and McCuaig, 2003; Tas et al., 2010; Durbin and Teske, 2011; Wasmund et al., 2015), and they potentially own versatile ecological functions such as CO_2_ fixation, dimethyl sulfoxide utilization, aromatics and fatty acids oxidization, and acetate production (Hug et al., 2013; Wasmund et al., 2014). The observed positive co-occurrence patterns within members of this class may suggest that these variable physiological features can be highly dependent and integrated, or help relieve interspecific competitions. The deep-abundant module was also involved in correlations among *Dehalococcoidia* and several other bacterial clades, including strictly anaerobic *Spirochaetaceae*, Sva0485 and low-oxygen-adapted candidate phylum OP8 (Bowman and McCuaig, 2003; Farag et al., 2014), *Pseudomonadaceae* and *Streptococcaceae*, implicating biogeochemical complexity in the deep marine sediments.

Comparatively, correlations among *Acidobacteria*, *Bacteroidetes*, *Proteobacteria*, and *Lentisphaerae* constituted the up-abundant module in the network, and the former three taxa were shown to have significantly higher proportions in the upper layers by LEfSe. JTB255-MBG, a member of order *Xanthomonadales* belonging to *Gammaproteobacteria*, was the most abundant clades (average 35%) in the upper layer sediments examined in this study. In the network, JTB255-MBG showed a high degree of connectivity with other surface-abundant taxa, indicating that growth of JTB255-MBG may be highly dependent on other bacterial clades, which may provide potential insights in developing new cultivating strategies for obtaining a pure isolate of this clade. Strains of *Lentisphaerae* were detected to produce transparent exopolymers (TEP) (Cho et al., 2004), a key factor of biofilm initiation and outgrowth (Berman and Passow, 2007). The involvement of *Lentisphaerae* and other marine biofilm residents, including *Rhodopirellula*, *Oceanospirillaceae*, *Alteromonadaceae*, *Acidobacteria, Planctomyces*, OM190, and *Bacteroidetes* (Bengtsson and Øvreås, 2010; Eichorst et al., 2011; Ruvindy et al., 2015; Lawes et al., 2016) in the up-abundant module indicated that biofilm may regulate bacterial interactions in the upper layer sediment.

## Conclusion

This study presented a detailed description of spatial and depth-related distribution patterns of bacterial communities in sediments of the ECMS. Abundance, diversity, and community structure varied significantly with sediment depth. The up and deep bacterial communities displayed different distribution patterns. The upper layer samples clustered in accordance with mud zones, whereas the deep layer samples of all sites tended to cluster together. TOC δ^13^C and TON δ^15^N significantly affected the bacterial community composition, suggesting that abundance and composition of organic matter played critical roles in shaping bacterial communities. Moreover, bacterial communities in the shallow and deep sediments showed different inter-taxa relationships, indicating different co-occurrence patterns in surface and subsurface sediments. This study provided a detailed outline of subsurface bacterial communities in mud deposits of the ECMS for the first time, and provided clues for uncovering biogeochemical cycles in this area.

## Author Contributions

YQ carried out sample collecting, laboratory work, data analysis, and drafted the manuscript. JL conceived the study, revised and finalized the manuscript. MZ and X-HZ participated in the design of the study and helped to draft the manuscript. All authors read and approved the final manuscript.

## Conflict of Interest Statement

The authors declare that the research was conducted in the absence of any commercial or financial relationships that could be construed as a potential conflict of interest.

## Footnotes

1 http://qiime.org

2 http://www.arb-silva.de
