# Supplementary material for: Sediment Depth-Dependent Spatial Variations of Bacterial Communities in Mud Deposits of the Eastern China Marginal Seas
Source: Front Microbiol. 2018 May 31;9:1128. doi: 10.3389/fmicb.2018.01128 (PMC5990616; doi:10.3389/fmicb.2018.01128)
Supplement: Supplementary file 1 [file Data_Sheet_1.PDF]

Supplementary Material

Sediment depth-dependent spatial variations of bacterial communities in mud deposits of the eastern China marginal seas

Yanlu Qiao[1,2], Jiwen Liu[1,2]*, Meixun Zhao[2,3], Xiao-Hua Zhang[1,2]

[1]Laboratory of Marine Microbiology, College of Marine Life Sciences, Ocean University of China, Qingdao, China

[2] Laboratory for Marine Ecology and Environmental Science, Qingdao National Laboratory for Marine Science and Technology, Qingdao, China

[3]Key Laboratory of Marine Chemistry Theory and Technology, Ministry of Education, Ocean University of China, Qingdao, China

*** Correspondence:**

Dr. Jiwen Liu

liujiwen@ouc.edu.cn

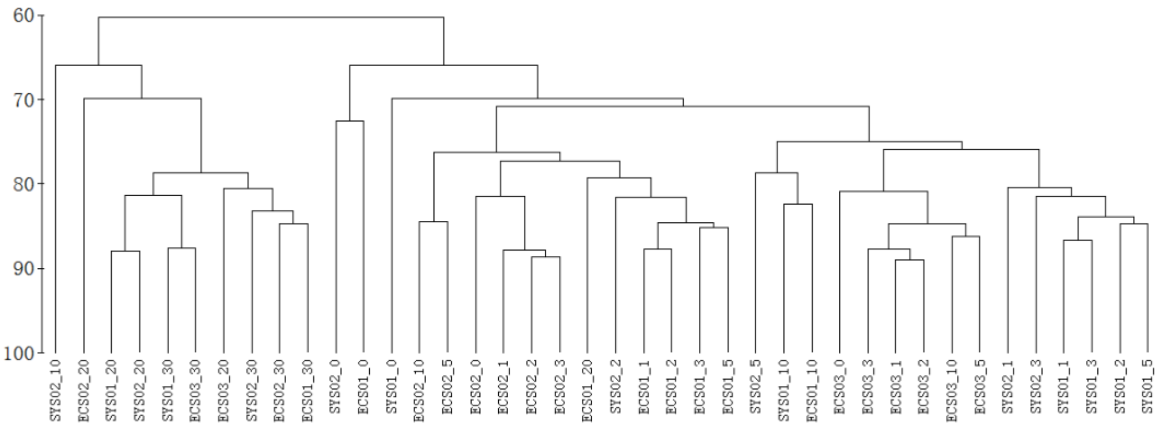

Figure S1 The Bray-Curtis similarity multisampling similarity dendrograms at the genus level.

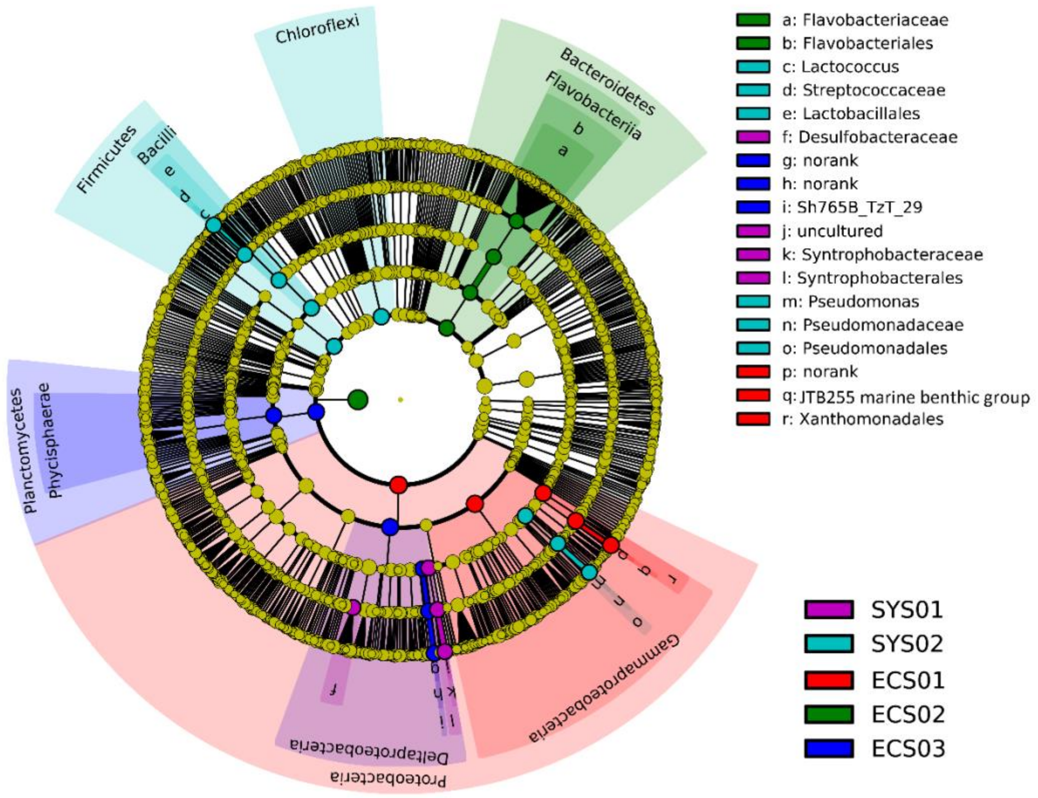

Figure S2 Cladograms showing the differences in abundance values between 5 sites according to LEfSe results with LDA threshold of 3.5. Taxa with significant differences were highlighted by colored circles and shadings.

Table S1 Observed bacterial richness and diversity estimates based on 97% OTU Uclusters

Sample ID	overlaped reads	filtered reads	rarefraction reads	OTU	chao1	shannon	coverage %
SYS01_0	42437	32119	24048	2865	3967	9.44	96.48
SYS01_1	33376	24491	24048	2601	3729	8.82	95.90
SYS01_2	23541	24048	24048	2987	4545	9.21	94.80
SYS01_3	37273	34347	24048	2679	4002	9.05	96.69
SYS01_5	40406	35480	24048	3087	4474	9.53	96.41
SYS01_10	36135	29786	24048	3324	4559	9.63	95.86
SYS01_20	34184	24861	24048	3135	4383	9.35	95.19
SYS01_30	44620	36576	24048	2216	2801	8.68	98.17
SYS02_0	33909	28987	24048	3247	4458	9.73	95.77
SYS02_1	34868	28899	24048	3628	5461	9.93	94.63
SYS02_2	44712	37033	24048	4020	5969	9.92	95.08
SYS02_3	36795	33977	24048	2729	3828	9.18	96.97
SYS02_5	38321	34010	24048	3167	4451	9.19	96.28
SYS02_10	36000	33309	24048	1788	2573	6.19	97.63
SYS02_20	31953	26520	24048	2610	3456	9.09	96.66
SYS02_30	38396	32590	24048	3760	5782	9.69	94.81
ECS01_0	32757	25861	24048	3243	4612	9.66	95.13
ECS01_1	41352	31748	24048	3419	4914	9.73	95.76
ECS01_2	33356	27245	24048	3231	4735	9.31	95.16
ECS01_3	36620	25922	24048	3656	5318	9.81	94.29
ECS01_5	34087	16890	24048	2928	3146	9.63	92.32
ECS01_10	44681	28600	24048	3468	5256	9.63	94.77
ECS01_20	35058	29225	24048	3144	4832	9.06	95.27
ECS01_30	35068	24860	24048	2867	4075	9.23	95.41
ECS02_0	31840	16844	24048	2971	3201	9.93	91.99
ECS02_1	39139	29897	24048	3392	4909	9.71	95.27
ECS02_2	37644	30907	24048	3208	4759	9.51	95.60
ECS02_3	35154	28150	24048	3337	4967	9.53	95.12
ECS02_5	32102	25886	24048	3179	4754	9.57	94.89
ECS02_10	44080	34435	24048	2698	4195	8.99	96.31
ECS02_20	31475	39416	24048	3380	5265	9.49	95.98
ECS02_30	39551	31865	24048	2834	3858	9.55	96.82
ECS03_0	35986	28125	24048	3575	5190	9.73	94.63
ECS03_1	33682	27775	24048	3793	5375	10.01	94.56
ECS03_2	34294	28986	24048	3633	5205	9.88	94.97
ECS03_3	42517	34726	24048	3360	4874	9.55	96.04
ECS03_5	43075	37664	24048	3676	5282	10.06	96.15
ECS03_10	34144	26884	24048	3561	5018	9.91	94.92
ECS03_20	33640	27887	24048	3629	5155	9.95	94.82
ECS03_30	44365	34025	24048	2590	3587	8.88	97.21
sum	1472593	1190856	961920				

Table S2 Correlations between percentage composition of taxa and environmental factors

	TOC%	TN%	C/N	δ^{13}C ‰	δ^{15}N ‰	N0$_3^-$	NO_2^-	NH_4^+	SiO_3^{2-}	PO_4^{3-}	depth
Acidobacteria											-0.482
Acidobacteria											-0.482
Subgroup 21	0.464										-0.420
Subgroup 9				0.479	0.421					**0.653**	
Actinobacteria		0.426					0.460				
Actinobacteria		0.426					0.460				
Bacteroidetes	**0.655**	**0.617**									-0.505
BD2-2	0.584	0.540									-0.471
Cytophagia		0.443			-0.517					-0.462	-0.555
Cytophagales		0.438			-0.495					-0.454	-0.533
Flammeovirgaceae		0.438			-0.487						-0.537
Flavobacteriia	**0.617**	0.470									
Flavobacteriales	**0.617**	0.470									
Flavobacteriaceae	**0.613**	0.457									
Sphingobacteriia					-0.591					-0.575	-0.464
Sphingobacteriales					-0.591					-0.575	-0.464
Candidate division BRC1					0.440				-0.475		
Candidate division OP3	-0.487	-0.537									0.506
Candidate division OP8											**0.657**
Chlorobi		0.423		-0.530							
Ignavibacteria				-0.555							
Ignavibacteriales				-0.555							
Chloroflexi											**0.652**
Ardenticatenia					-0.456						
Dehalococcoidia											**0.656**
vadinBA26											0.592
Firmicutes			0.527								
Bacilli			0.526								
Lactobacillales			0.527								
Streptococcaceae			0.526								
Lactococcus			0.525								
Gemmatimonadetes											
Gemmatimonadetes											

	TOC%	TN%	C/N	δ13C ‰	δ15N ‰	$N0_3^-$	NO_2^-	NH_4^+	SiO_3^{2-}	PO_4^{3-}	depth
BD2-11 terrestrial group			0.484	**-0.613**	**-0.693**					-0.560	
Lentisphaerae		0.434			-0.426					-0.437	
Nitrospirae	**-0.643**	-0.524									
Nitrospira	**-0.643**	-0.524									
Planctomycetes					0.515						
OM190											**-0.702**
Phycisphaerae					0.539						
MSBL9											0.528
Phycisphaerales	-0.421			0.537							
Phycisphaeraceae				0.443					0.497		
Urania-1B-19	-0.425			0.478					0.522		
Pla3 lineage			-0.579							-0.504	
Planctomycetacia	0.514	0.525									**-0.638**
Planctomycetales	0.512	0.526									**-0.637**
Planctomycetaceae	0.512	0.526									**-0.637**
Pir4 lineage	0.542				0.490					0.547	
Planctomyces	**0.640**	**0.609**				0.456					**-0.624**
Rhodopirellula											**-0.753**
Proteobacteria											-0.418
Rhodobacterales		0.466									
Rhodobacteraceae		0.466									
Rhodospirillales											-0.468
Rhodospirillaceae											-0.455
Betaproteobacteria				-0.585	-0.524					-0.501	
Deltaproteobacteria					0.552						
Desulfarculales		-0.439									**0.706**
Desulfarculaceae		-0.439									**0.706**
Desulfobulbaceae					-0.441						
Desulfobulbus					-0.458						-0.452
Desulfuromonadales											-0.489
Myxococcales											-0.550
Sandaracinaceae							0.481				-0.506
Sh765B-TzT-29				0.449	0.489						
Sva0485											**0.665**
Syntrophobacterales					**0.620**					0.511	

	TOC%	TN%	C/N	δ13C ‰	δ15N ‰	N0$_3^-$	NO$_2^-$	NH$_4^+$	SiO$_3^{2-}$	PO$_4^{3-}$	depth
Syntrophobacteraceae					**0.618**					0.511	
Gammaproteobacteria					**-0.667**						
Alteromonadales									0.460		
Alteromonadaceae									0.456		
BD7-8 marine group										-0.455	-0.444
Chromatiales			-0.546		**-0.755**						
Ectothiorhodospiraceae			-0.440	-0.504	**-0.863**						
Acidiferrobacter			-0.448	-0.507	**-0.870**						
KI89A clade											-0.453
Order Incertae Sedis		0.466			-0.418						-0.539
Family Incertae Sedis		0.466			-0.418						-0.539
Marinicella		0.466			-0.418						-0.539
Pseudomonadales			0.520								
Moraxellaceae			0.522								
Pseudomonadaceae			0.518								
Pseudomonas			0.518								
Xanthomonadales				-0.467	**-0.671**			0.551			
JTB255				-0.462	**-0.670**			0.540			
Spirochaetae	-0.459	-0.512									**0.623**
Spirochaetes	-0.459	-0.512									**0.623**
Spirochaetales	-0.509	-0.538									**0.606**
Spirochaetaceae	-0.496	-0.529									**0.611**
Spirochaeta	-0.505	-0.510									0.543
TM6											0.450

Only significant Correlation (*P*<0.01) were shown in table.

Supplementary script R script of network analysis

```
library(igraph)
library(Hmisc)
ar<-read.table(file="clipboard",header=F)
rownames(ar)<-ar[,1]
ar<-ar[,-1]
arr<-rcorr(t(ar),type="spearman")
p.adjust.vector<-p.adjust (arr$P, method="BH") # Control the false discovery rate
dim(arr$P) #x,x
p.adjust.matrix<-matrix(p.adjust.vector,nc=x,nr=x)
rownames(p.adjust.matrix)<-rownames(ar)
colnames(p.adjust.matrix)<-rownames(ar)
r<-arr$r
q<-p.adjust.matrix
q[is.na(q)]<-0
for(i in 1:x)
{
 for(j in 1:x)
  {
   if ( (abs(r[i,j]) > 0.7) & (q[i,j] < 0.01) )
     { r[i,j]=r[i,j] }
   else
     {    r[i,j]=0   }
```

```
  }
}
a.graph<-graph.adjacency(r, weight=T, mode="undirected")
a.graph<-simplify(a.graph)
is.simple(a.graph)
V(a.graph)$phylum <- phylum
V(a.graph)$degree <- degree(a.graph)
write.graph(a.graph, "net.gml", format='gml')
```
